# Functional characterization of UHRF1 variants in facilitating DNA methylation

**DOI:** 10.1016/j.jbc.2026.113149

**Published:** 2026-05-14

**Authors:** Bigang Liu, Kaila Nayvelt, Swanand Hardikar, Kimie Kondo, Marcos R. Estecio, Xiaodong Cheng, Taiping Chen

**Affiliations:** 1 Department of Epigenetics and Molecular Carcinogenesis, The University of Texas MD Anderson Cancer Center, Houston, Texas, USA; 2 Program in Genetics and Epigenetics, The University of Texas MD Anderson Cancer Center UTHealth Graduate School of Biomedical Sciences, Houston, Texas, USA

**Keywords:** 5-methylcytosine, DNA methylation, DNA methyltransferase, embryonic stem cell, epigenetics, epitope tag, ICF syndrome, UHRF1

## Abstract

Ubiquitin-like with plant homeodomain (PHD) and really interesting new gene (RING) finger domains 1 (UHRF1) is essential for DNA methylation inheritance. However, the functional impacts of several natural and engineered UHRF1 variants are either insufficiently characterized or obscured by conflicting results, with some discrepancies likely stemming from cellular toxicity and adaptive responses induced by DNA methylation changes. In this study, we utilized mouse embryonic stem cells (mESCs)—which uniquely tolerate the complete loss of DNA methylation—to evaluate the functional consequences of clinical mutations, isoform variation, and epitope tagging. Using rescue experiments in *Uhrf1*-deficient mESCs, we characterized two UHRF1 mutations identified in a patient with immunodeficiency, centromeric instability, and facial anomalies (ICF) syndrome, demonstrating that the R618X nonsense mutation creates a null allele, while the R296W missense mutation is hypomorphic. Furthermore, we confirmed that N-terminal tagging abolishes UHRF1 function, whereas human UHRF1 (hUHRF1) isoform 2, featuring a 13-residue N-terminal extension, is functionally inactive. AlphaFold3 structural predictions suggest that these additional residues at the N terminus disrupt essential inter-domain interactions. Collectively, our results define the activity of UHRF1 variants and resolve existing inconsistencies in the field.

DNA methylation—the addition of a methyl group to the C-5 position of cytosine, generating 5-methylcytosine (5mC)—is a crucial eukaryotic epigenetic modification involved in a variety of cellular and developmental processes. In mammals, DNA methylation occurs predominantly at CpG dinucleotides to form symmetrical patterns, which are semi-conservatively maintained across cell divisions. While the *de novo* DNA methyltransferases 3A (DNMT3A) and 3B (DNMT3B) are primarily responsible for establishing DNA methylation patterns (1, 2), DNMT1, which prefers hemi-methylated CpG sites, is the major enzyme for maintaining these patterns upon DNA replication (3, 4, 5, 6). The maintenance process requires the essential accessory factor ubiquitin-like with plant homeodomain (PHD) and really interesting new gene (RING) finger domains 1 (UHRF1) (7, 8).

UHRF1 is a multi-domain protein featuring five conserved regions: an N-terminal ubiquitin-like (UBL) domain, followed by a tandem Tudor domain (TTD), a PHD, a SET and RING-associated (SRA) domain, and a C-terminal RING domain. These domains coordinate to recruit DNMT1 to newly synthesized DNA. Central to this process, the SRA domain recognizes hemi-methylated DNA and interacts with DNMT1, facilitating DNMT1 loading and inducing DNMT1 activity (7, 8, 9, 10, 11, 12, 13). The TTD and PHD cooperatively bind histone H3 and DNA ligase 1 (LIG1) with specific lysine and arginine methylation patterns (14, 15, 16, 17, 18, 19). Additionally, the RING domain acts as an E3 ubiquitin ligase—aided by UBL domain-mediated E2 recruitment—to catalyze monoubiquitination at specific lysine residues in histone H3 and proliferating cell nuclear antigen (PCNA)-associated factor 15 (PAF15), creating binding sites for DNMT1 (20, 21, 22, 23, 24, 25).

Faithful inheritance of DNA methylation is vital for cellular homeostasis. Genetic ablation of *Dnmt1* or *Uhrf1* in mice results in mid-gestational embryonic lethality (4, 8). Aberrant DNA methylation patterns are associated with various human diseases, with a prominent example being immunodeficiency, centromeric instability, and facial anomalies (ICF) syndrome, a rare antibody deficiency disorder characterized by hypomethylation of DNA repeats in centromeric/pericentromeric regions (26). The vast majority of ICF patients carry biallelic mutations in one of four genes: *DNMT3B* (ICF1), zinc finger- and BTB domain-containing 24 (*ZBTB24*, ICF2), cell division cycle associated 7 (*CDCA7*, ICF3), and helicase, lymphoid-specific (*HELLS*, ICF4) (2, 27, 28, 29, 30). Comparative methylation profiling clearly distinguishes ICF1 from ICF2-4, despite common methylation alterations (31). Emerging evidence suggests that ZBTB24, CDCA7, and HELLS form a regulatory pathway that primarily facilitates the maintenance, rather than establishment, of DNA methylation patterns at specific heterochromatic regions (32, 33, 34, 35, 36, 37, 38). In support of this notion, a recent paper reported an atypical case of ICF syndrome caused by compound heterozygous mutations in *UHRF1*, one allele carrying a missense mutation, R296W, in the linker region between TTD and PHD, and the other allele carrying a nonsense mutation, R618X, that truncates the C-terminal 176 amino acid residues, including the RING domain (39) (Fig. 1*A*).

**Figure 1:**
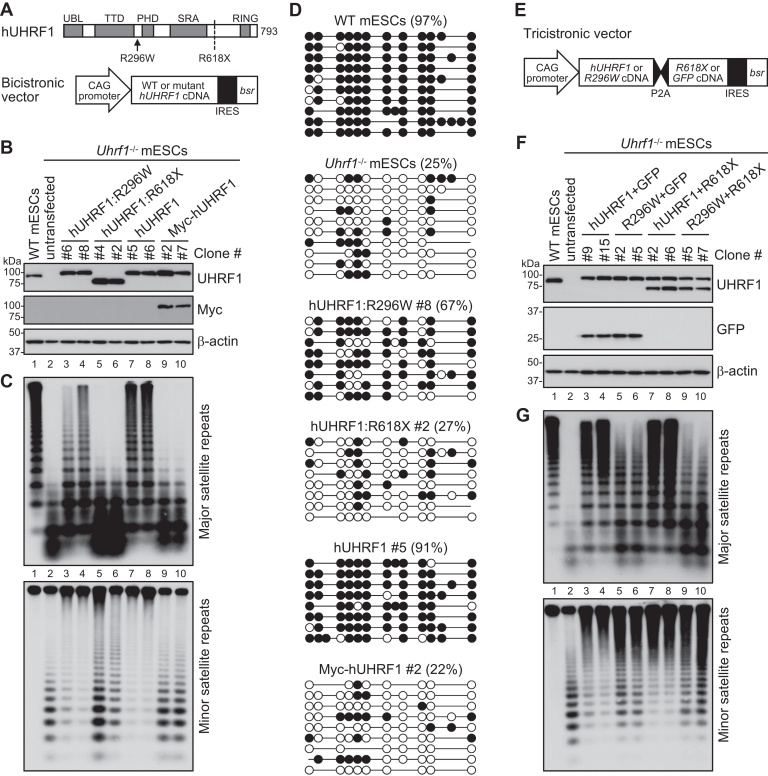
**The UHRF1 R296W and R618X mutations are partial and complete loss-of-function mutations, respectively.***A*, *top*: hUHRF1 protein with the five functional domains. The locations of the missense mutation, R296W, and the nonsense mutation, R618X, associated with ICF syndrome are indicated. *Bottom*: Schematic diagram of the IRES-containing bicistronic plasmid vector used to express WT or mutant hUHRF1 and the blasticidin S-resistance (*bsr*) gene (selection marker) from the same transcript. *B*, Western blots showing the expression of mutant or WT (untagged or Myc-tagged) hUHRF1 proteins in stable clones established in *Uhrf1*^*−/−*^ mESCs. Shown from *top* to *bottom* are blots with UHRF1, Myc tag, and β-actin antibodies. *C*, Southern blots showing DNA methylation levels at the major and minor satellite repeats in the same cell lines shown in *panelB*. *D*, Bisulfite sequencing analysis showing DNA methylation levels in a region of IAP retrotransposons. For each sample, eight to 10 clones were sequenced. Note that the amplified region contains 8 to 13 CpG dinucleotides due to sequence variations. Unmethylated and methylated CpG sites are shown as open and filled circles, respectively. The methylation percentage in each sample is indicated. *E*, schematic diagram of the tricistronic plasmid vector used to express the selection marker *bsr* gene and two additional proteins (protein 1: WT hUHRF1 or the R296W mutant; protein 2: the R618X mutant or GFP) by including the “self-cleaving” P2A peptide. *F*, Western blots showing the expression of different combinations of proteins in stable clones established in *Uhrf1* KO mESCs. Shown from top to bottom are blots with UHRF1, GFP, and β-actin antibodies. *G*, Southern blots showing DNA methylation levels at the major and minor satellite repeats in the same cell lines shown in panel *F*.

While the role of UHRF1 in DNA methylation is well-established, several of its naturally occurring and experimentally engineered variants have either been understudied or yielded inconsistent data. Here, we show that the ICF mutant R296W acts as a hypomorph, while R618X creates a null allele. Furthermore, we confirm that N-terminal tagging inactivates UHRF1, aligning with two previous reports (24, 25) but contradicting numerous other studies (16, 20, 40, 41, 42). Additionally, we demonstrate that human UHRF1 (hUHRF1) isoform 2, characterized by a 13-residue N-terminal extension, lacks activity in regulating DNA methylation.

## Results

### Characterization of *UHRF1* mutations associated with ICF syndrome

To assess the functional effects of hUHRF1 ICF mutations, R296W and R618X (Fig. 1*A*), we compared WT and mutant hUHRF1 proteins for their abilities to rescue DNA methylation in *Uhrf1* knockout (KO) (*Uhrf1*^*−/−*^) mouse embryonic stem cells (mESCs) (43). cDNAs encoding WT hUHRF1 [untagged or N-terminally Myc-tagged (Myc-hUHRF1)], hUHRF1:R296W, and hUHRF1:R618X (*i.e.*, amino acids 1–617) were cloned into a bicistronic plasmid vector containing an internal ribosome entry site (IRES)-driven blasticidin S-resistance (*bsr*) selection cassette (44) (Fig. 1*A* and Table S1). The plasmids were transfected into *Uhrf1*^*−/−*^ mESCs, stable clones were obtained following selection with blasticidin S, and those expressing WT or mutant hUHRF1 proteins at similar levels were used for experiments (Fig. 1*B*).

Repetitive elements in the genome are abundant, widely distributed, and highly methylated, and their methylation can serve as a proxy for global DNA methylation. We first analyzed the methylation levels at minor satellite repeats in centromeric regions and major satellite repeats in pericentromeric regions by Southern hybridization following digestion of genomic DNA with methylation-sensitive restriction enzymes. In this semi-quantitative assay, the sizes of the DNA fragments correlate with methylation levels. As expected, *Uhrf1*^*−/−*^ mESCs were severely hypomethylated (Fig. 1*C*, lane 2) compared to WT mESCs (lane 1). When expressed in *Uhrf1*^*−/−*^ mESCs, hUHRF1:R296W restored DNA methylation to a substantial extent (lanes 3–4), but the rescue effect was not as strong as that of WT hUHRF1 (lanes 7–8). By contrast, the truncated protein product of hUHRF1:R618X showed no remethylation activity (lanes 5–6), consistent with the previous finding that the RING domain is essential for UHRF1 function (20, 21, 45). Intended as an additional “positive” control, N-terminally tagged Myc-hUHRF1 was included in the experiment, but it turned out to be inactive in rescuing DNA methylation in *Uhrf1*^*−/−*^ mESCs (lanes 9–10), unlike the untagged version of hUHRF1 (lanes 7–8).

We also performed bisulfite sequencing analysis, a quantitative assay, to measure the CpG methylation levels of a region in intracisternal A-particle (IAP) retrotransposons. The results confirmed that hUHRF1:R618X and Myc-hUHRF1 were completely inactive, while hUHRF1:R296W was modestly impaired, with its activity being estimated at 60 to 70% of the level of WT hUHRF1 $\left(\text{Relative}\;\text{activity}=\frac{R296W-\text{Uhrf}1\;\text{KO}}{\text{WT}-\text{Uhrf}1\;\text{KO}}=\frac{67-25}{91-25}=64\%\right)$ (Fig. 1*D*).

Next, we asked whether the truncated protein from the nonsense mutant R618X, albeit inactive by itself (Fig. 1, *C* and *D*), would interfere with the activity of WT hUHRF1 or the R296W mutant protein. To test this potential “dominant-negative” effect, we established stable clones in *Uhrf1*^*−/−*^ mESCs coexpressing full-length hUHRF1 (WT or R296W mutant) and the truncated hUHRF1 protein (1–617 amino acids) or GFP (negative control) from a tricistronic plasmid vector that contains the sequence encoding the “self-cleaving” 2A peptide of porcine teschovirus-1 (P2A) (Fig. 1*E*). As expected, immunoblotting results suggested equal levels of expression of both proteins in each clone (Fig. 1*F*). As shown in Figure 1*G*, coexpression of the truncated hUHRF1 protein, relative to GFP, had no effect on the remethylation activities of both WT hUHRF1 (compare lanes 3–4 with 7–8) and the R296W mutant protein (compare lanes 5–6 with 9–10). In summary, our data demonstrate that hUHRF1:R296W is a hypomorphic mutation and hUHRF1:R618X a null mutation with no dominant-negative effect on DNA methylation.

### N-terminally tagged mUHRF1 is unable to rescue DNA methylation in *Uhrf1*^*−/−*^ mESCs

**Figure 2:**
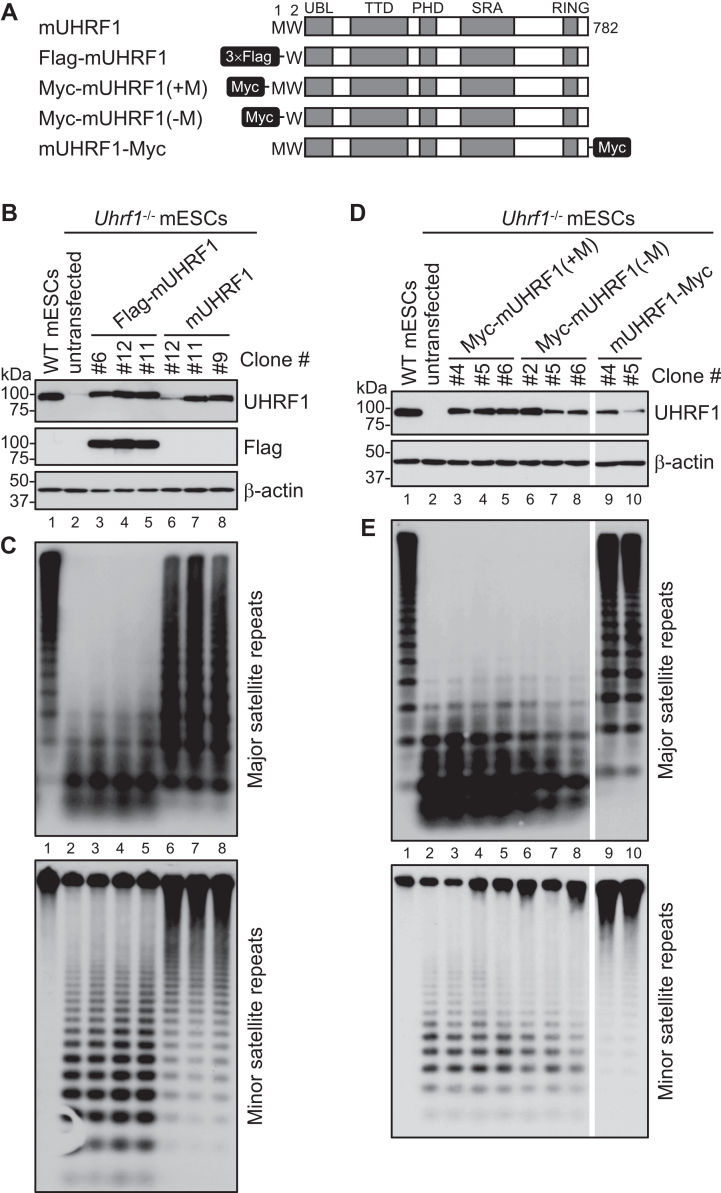
**N-terminally tagged mUHRF1 fails to rescue DNA methylation in *Uhrf1*^*−/−*^ mESCs.***A*, schematic diagram of the untagged and tagged mUHRF1 proteins used in this figure. The first two amino acids (MW) are shown. Note that the start codon of mUHRF1 is deleted in Flag-mUHRF1 and Myc-mUHRF1(−M) but present in untagged, Myc-mUHRF1(+M) and mUHRF1-Myc. *B and D*, Western blots showing the expression of mUHRF1 proteins in stable clones established in *Uhrf1* KO mESCs. *C* and *E*, Southern blots showing DNA methylation levels at the major and minor satellite repeats in the same cell lines shown in *panelsB* and *D*, respectively. In each blot of panels *D* and *E*, the *left* (lanes 1–8) and *right* (lanes 9–10) parts were from the same blot (with several lanes in-between being removed).

Consistent with our observation that Myc-hUHRF1 is inactive (Fig. 1, *C* and *D*), two previous studies reported that N-terminally tagged UHRF1 fails to restore DNA methylation in *Uhrf1*^*−/−*^ mESCs (24, 25). However, N-terminally tagged hUHRF1 and mouse UHRF1 (mUHRF1) have been used without reported adverse effects by numerous other studies (16, 20, 40, 41, 42). To resolve this inconsistency, we stably expressed various versions of mUHRF1 in *Uhrf1*^*−/−*^ mESCs and evaluated their rescue capability (Fig. 2*A* and Table S1).

We first demonstrated that N-terminally 3×Flag-tagged mUHRF1 lacks remethylation activity (Fig. 2, *B* and *C*, lanes 3–5). Because translation initiates at the 3×Flag tag, this construct lacks the mUHRF1 initiator methionine (M1) (Fig. 2*A*). To determine whether M1 removal caused this inactivation, we tested N-terminally Myc-tagged mUHRF1 with and without M1 [Myc-mUHRF1(+M) and Myc-mUHRF1(−M), respectively (Fig. 2*A*)]; both proved inactive (Fig. 2, *D* and *E*, lanes 3–8). Conversely, C-terminally tagged mUHRF1 (mUHRF1-Myc, Fig. 2*A*) was fully functional (Fig. 2, *D* and *E*, lanes 9–10). Notably, clones with low expression of untagged mUHRF1 (clone #12) or mUHRF1-Myc (clone #5) achieved methylation levels similar to high-expressing clones (Fig. 2, *B–E*), indicating that low levels of UHRF1 are sufficient for activity.

### UHRF1 function correlates with N-terminal extension length

**Figure 3:**
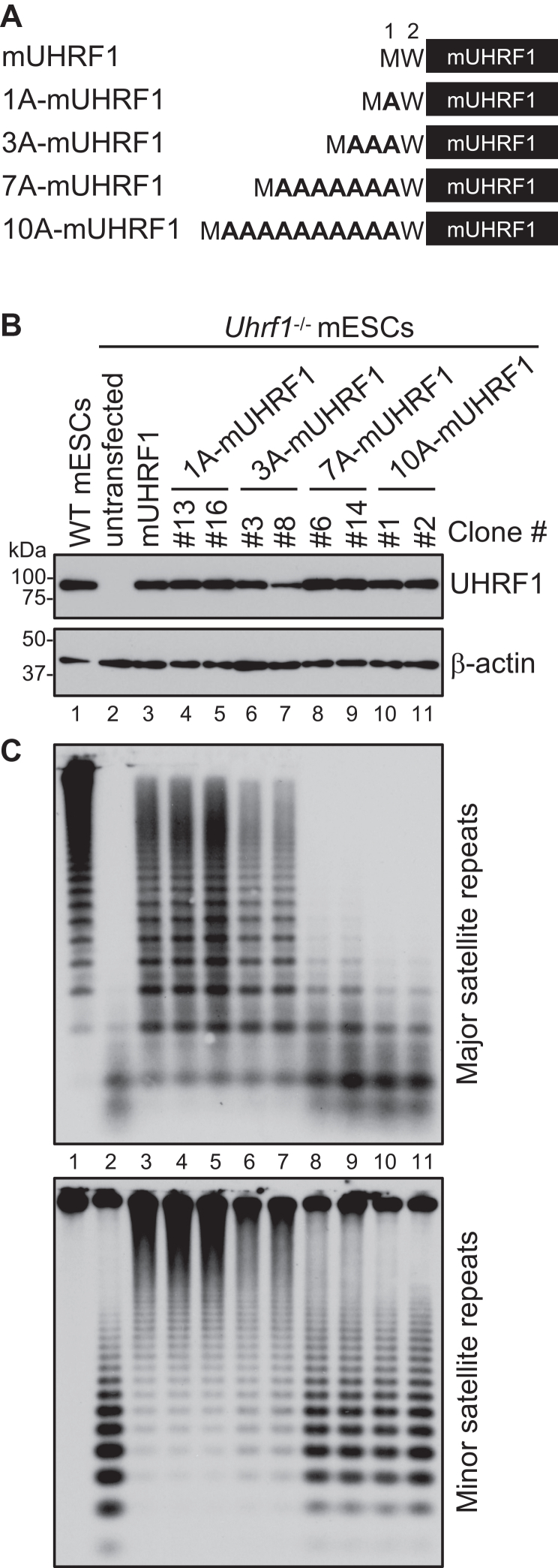
**The effect on UHRF1 function correlates with the length of N-terminal extension.***A*, schematic diagram of the mUHRF1 proteins used in this figure. The extra alanine residues in 1A-, 3A-, 7A- and 10A-mUHRF1 were added after the start codon. *B*, Western blots showing the expression of mUHRF1 proteins in stable clones established in *Uhrf1* KO mESCs. *C*, Southern blots showing DNA methylation levels at the major and minor satellite repeats in the same cell lines shown in *panelB*.

Our results demonstrate that UHRF1 is completely inactivated by the addition of a Myc tag (10 residues) or a 3×Flag tag (22 residues) at its N terminus (Figs. 1 and 2). Since some of the commonly used epitope tags are smaller [*e.g.*, Flag (8 residues) and HA (9 residues)], we investigated the effect of N-terminal extension length by adding varying numbers of alanine (A) residues to the N terminus of mUHRF1 (Fig. 3*A* and Table S1). Rescue experiments in *Uhrf1*^*−/−*^ mESCs revealed a clear negative correlation between mUHRF1 activity and the number of residues added (Fig. 3, *B* and *C*). Relative to the remethylation activity of untagged mUHRF1 (lane 3), addition of a single residue (1A-mUHRF1) had no effect (lanes 4–5), but addition of three (3A-mUHRF1, lanes 6–7) and seven residues (7A-mUHRF1, lanes 8–9) resulted in modest and severe impacts, respectively, with 10 residues (10A-mUHRF1) causing almost complete inactivation (lanes 10–11). While our data indicate that the length of the N-terminal extension is the primary determinant of UHRF1 activity, the fact that 10A-mUHRF1 retained residual activity (Fig. 3*C*), whereas Myc-mUHRF1 and Myc-hUHRF1 (also with 10 additional residues) showed no detectable activity (Figs. 1 and 2), suggesting that the sequence of the N-terminal extension also influences function.

### hUHRF1 isoform 2, featuring a 13-residue N-terminal extension, is inactive

**Figure 4:**
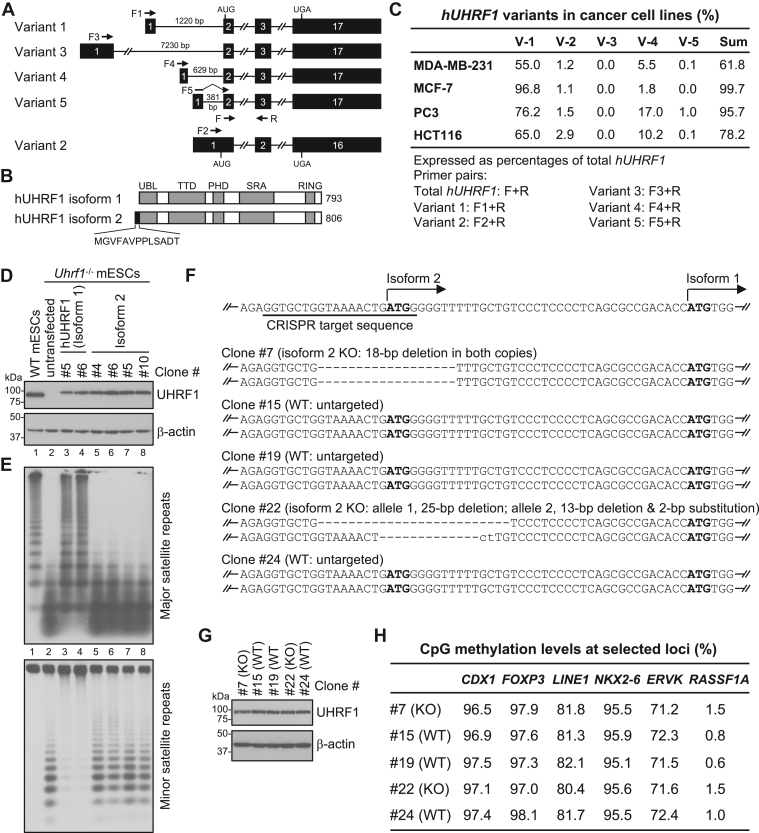
**hUHRF1 isoform 2 is inactive in regulating DNA methylation.***A*, schematic representation of how the five *hUHRF1* transcript variants are generated. Note that variant 2, with retention of the first intron of variant 5, comprises 16 exons, whereas variant 5 and the other variants comprise 17 exons. The locations of the start (AUG) and stop (UGA) codons and the primers used for RT-qPCR analysis (data shown in *panelC*) are indicated. Note that the variant 5-specific primer, F5, consists of sequences at the end of exon 1 and the beginning of exon 2. *B*, hUHRF1 protein products. Isoform 1 (canonical hUHRF1) is encoded by variants 1, 3, 4, and 5, and isoform 2, with 13 additional amino acid residues at the N terminus, is encoded by variant 2. *C*, RT-qPCR analysis showing the relative levels of *hUHRF1* variants in four cancer cell lines. Shown are percentages of the variants in total *hUHRF1* ($\text{variant}\;\text{percentage}=\frac{\text{variant}\;\text{level}}{\text{total}\;hUHRF1\;\text{level}}\times 100\%$). Sum = V1+V2+V3+V4+V5. *D*, Western blots showing the expression of hUHRF1 isoform 1 or isoform 2 in stable clones established in *Uhrf1* KO mESCs. Note that hUHRF1 isoform 1 (793 aa) migrated more slowly than endogenous mUHRF1 (782 aa), as expected, whereas hUHRF1 isoform 1 (793 aa) and isoform 2 (806 aa) showed no difference in migration (even after long electrophoresis), likely reflecting the influence of structural changes in isoform 2 (see Fig. 5). *E*, Southern blots showing DNA methylation levels at the major and minor satellite repeats in the same cell lines shown in *panelD*. *F*, generation of hUHRF1 isoform 2-specific KO HCT116 cells by CRISPR/Cas9 gene editing. Shown at the *top* is the targeting strategy, with the CRISPR target sequence being underlined. Shown below are the sequencing results of two KO clones (#7 and #22), with deletion of the isoform 2 start codon in both copies, and three clones (#15, #19 and #24) with no indels (untargeted). The start codons (ATG) of the two hUHRF1 isoforms are indicated. Deletion of a nucleotide is shown as a hyphen (−), and nucleotide substitutions are shown in lowercase letters. *G*, Western blots showing similar levels of hUHRF1 in isoform 2 KO and WT HCT116 cells. *H*, pyrosequencing methylation analysis of five loci showing that deletion of hUHRF1 isoform 2 has no effects on DNA methylation.

Based on GenBank records, *hUHRF1* transcription initiates at multiple sites, yielding at least five distinct transcript variants [Accession numbers: NM_001048201.3 (variant 1), NM_013282.5 (variant 2), NM_001290050.2 (variant 3), NM_001290051.2 (variant 4), and NM_001290052.2 (variant 5)]. Variants 1, 3, 4, and 5 utilize a shared translation start codon in exon 2, generating the canonical 793-residue protein (isoform 1). Variant 2, which shares the transcription start site with variant 5, retains the first 381-bp intron of variant 5 (Fig. 4*A*). This retention enables translation initiation at an upstream in-frame start codon, producing isoform 2, which contains a 13-residue N-terminal extension (Fig. 4*B*).

Comparison of ape genome sequences (46) suggests that UHRF1 isoform 2 (with an identical N-terminal extension) is conserved in humans’ closest relatives—chimpanzees, bonobos, and gorillas. However, this isoform is not present in distant primates like orangutans and siamangs nor is it found in rodents (Fig. S1*A*). GenBank records show that *mUhrf1* produces at least eight transcript variants encoding five protein products (isoforms A-E), none of which have an N-terminal extension (Fig. S1, *B* and *C*).

To evaluate the expression of *hUHRF1* variants, we performed RT-qPCR analysis using variant-specific primers (Table S2) in four cancer cell lines: MDA-MB-231 (breast cancer), MCF-7 (breast cancer), PC3 (prostate cancer), and HCT116 (colon cancer). Expression patterns were similar in all the cell lines. Among the total *hUHRF1* transcripts, analyzed by a primer pair common for all variants (Fig. 4*A* and Table S2), variant 1 is the predominant isoform, constituting ∼55 to 97%, followed by variant 4 (∼2–17%), while variants 3 and 5 are barely detectable, and variant 2 is expressed at low levels (∼1–3%) (Fig. 4*C*). The sum of variant percentages is substantially lower than 100% in MDA-MB-231 (∼62%) and HCT116 (∼78%) (Fig. 4*C*), implying the possible presence of additional variants in these cell lines. Indeed, GenBank records include several predicted *hUHRF1* variants (variants X1-X4) by automated computational analysis.

Having observed that N-terminal extensions impair UHRF1 function (Figure 1, Figure 2, Figure 3), we assessed the activity of hUHRF1 isoform 2. Rescue experiments demonstrated that, unlike canonical isoform 1, isoform 2 failed to induce remethylation in *Uhrf1*^*−/−*^ mESCs (Fig. 4, *D* and *E*, compare lanes 3–4 with 5–8). To examine the impact of isoform 2 loss, we performed CRISPR/Cas9 gene editing in HCT116 cells. We obtained two isoform 2 KO clones (#7 and #22) with biallelic deletions covering the translation start codon, along with three control (WT) clones (#15, #19, and #24) with no indels (Fig. 4*F*). Immunoblotting revealed comparable levels of hUHRF1 in all the clones (Fig. 4*G*), indicating that isoform 1 expression was unaffected in isoform 2 KO cells. Both isoforms migrate at the same rate (Fig. 4*D*), precluding confirmation of isoform 2 disappearance in the KO clones. Pyrosequencing methylation analysis revealed that isoform 2 ablation in HCT116 cells exerted no positive or negative effect on DNA methylation, regardless of whether the regions were highly methylated [caudal type homeobox 1 (*CDX1*), forkhead box protein P3 (*FOXP3*), and NK2 homeobox 6 (*NKX2-6*) promoters], partially methylated [long interspersed nuclear element 1 (*LINE1*) and endogenous retrovirus-K (*ERVK*) retrotransposons], or unmethylated [Ras association domain family 1 isoform A (*RASSF1A*) promoter] (Fig. 4*H*). The isoform 2 KO cells also exhibited no changes in morphology, viability, and proliferation.

**Figure 5:**
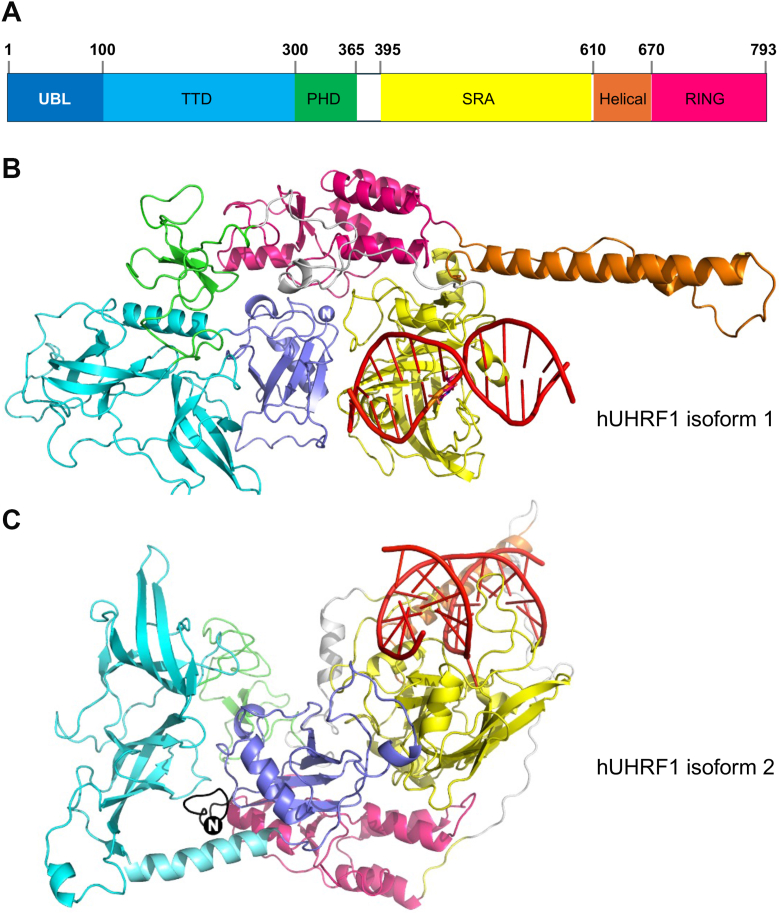
**AlphaFold3 predicts strikingly different conformations for hUHRF1 isoforms 1 and 2.***A*, domain architecture of hUHRF1 isoform 1, color-coded from the N- to C-termini: UBL (*dark blue*), TTD (*light blue*), PHD (*green*), linker (*grey*), SRA (*yellow*), helical domain (*orange*), and RING (*red*). *B*, alphaFold3 model of hUHRF1 isoform 1 bound to DNA. The N-terminal UBL domain is centrally positioned and surrounded by the TTD on the left, the PHD and RING domain above, and the DNA-bound SRA domain on the right. The only domain that does not directly contact the UBL is the helical region between the SRA and RING domains. The first methionine is shown as a sphere (labeled as “N”) and is buried within the core of the molecule. *C*, in isoform 2, the additional N-terminal residues (shown in *black*) are exposed on the surface and subsequently alter the overall domain arrangement relative to isoform 1. The AlphaFold3 models show relatively high confidence for structured domains, with predicted local distance difference test (pLDDT) values ranging from 70 to 90 (on a 0–100 scale), whereas interdomain regions and loops display lower confidence (pLDDT = 50–70). The predicted template modeling (pTM) score, reflecting overall structural accuracy, is 0.81 for isoform 1 and 0.85 for isoform 2. The interface predicted TM (ipTM) score is 0.98 for both isoforms, indicating high confidence in domain–domain interactions.

To understand how the N-terminal extension in isoform 2 renders hUHRF1 inactive, we utilized AlphaFold3 (47) to predict the structures of both isoforms. In isoform 1, the N-terminal UBL domain is centrally positioned, which coordinates with the other functional domains (*i.e.*, TTD, PHD, SRA, and RING domains) (Fig. 5, *A* and *B*). In isoform 2, the additional N-terminal residues cannot be accommodated in the center of the structure, forcing a rearrangement of intra-molecule interactions and causing drastic structural changes (Fig. 5*C*).

## Discussion

As a stable epigenetic modification, DNA methylation plays crucial roles in establishing and maintaining cell identity. Most cell types, including cancer cell lines, are intolerant to significant changes in DNA methylation, and cellular toxicity and adaptive responses may have contributed to inconsistent UHRF1 data in previous literature. mESCs are unique in that their survival and proliferation are unaffected by the complete loss of DNA methylation (48), offering a major advantage for studying DNA methylation regulators. Our rescue experiments in *Uhrf1*^*−/−*^ mESCs clearly demonstrate that: a) the ICF mutation R618X creates a functional null allele, while the R296W mutation results in a hypomorphic allele; b) N-terminally tagged UHRF1 lacks activity; and c) the 13-residue N-terminal extension renders hUHRF1 isoform 2 inactive. AlphaFold3 predictions suggest that N-terminal extensions disrupt essential interactions between the UBL domain and the other functional domains, leading to significant conformational changes.

Our data reveal that the hUHRF1:R296W mutation is relatively mild, retaining 60% to 70% of WT activity. This residue is notably not conserved in mUHRF1 (Fig. S2). These results rationalize the modest clinical phenotype of the reported ICF patient, who lacked immunodeficiency and displayed only moderate hypomethylation of classical satellite-2 DNA (39, 49). The mild effect also helps explain why HEK293 cells expressing the mutant protein in the absence of endogenous UHRF1 do not exhibit hypomethylation until after one month of continuous culturing (39). Given that mice heterozygous for a *Uhrf1* null allele are phenotypically normal (8), the pathogenesis of the atypical ICF syndrome may not be simply due to haploinsufficiency. It appears that the hypomorphic allele supports survival but is insufficient for normal development and physiology.

Two previous studies indicated, without showing experimental data, that N-terminally tagged UHRF1 is inactive or less effective in restoring DNA methylation in *Uhrf1*^*−/−*^ mESCs (24, 25). This important observation has not been widely recognized, as N-terminally tagged UHRF1 is still utilized in recent work (42). Our results show that the deleterious effect correlates with N-terminal extension length: a 7-residue extension severely impairs UHRF1 activity, while 10 or more residues abolish it. Given that commonly used epitope tags exceed seven residues, we predict little to no activity for all such N-terminally tagged UHRF1 versions. Consequently, results and conclusions from studies using such constructs warrant reevaluation.

Finally, we find that UHRF1 isoform 2—present only in humans and other hominines (chimpanzee, bonobo, and gorilla)—is inactive in restoring DNA methylation in *Uhrf1*^*−/−*^ mESCs and that its ablation in HCT116 cells has no effect on DNA methylation. While our findings indicate that isoform 2 does not play a role in regulating DNA methylation, it remains to be determined whether it serves methylation-independent functions—potentially through its histone-binding activity and the E3 ligase activity of the RING domain—which will require further experimental validation, particularly as full-length structural approaches become feasible.

## Experimental procedures

In this study, we stably expressed various versions of hUHRF1 and mUHRF1 in *Uhrf1*^*−/−*^ mESCs and assessed their capabilities of restoring DNA methylation. The Western and Southern blots shown in each figure panel were repeated at least twice with multiple individual clones, which showed highly consistent results. Representative data are presented. Described below are the materials, methods, and experimental procedures used.

### Plasmid vectors

The plasmid vectors used for protein expression were generated by cloning *hUHRF1*, *mUhrf1* or *GFP* cDNAs into *pCAG-3×Flag-IRESblast* or *pCAG-3*×*Flag-P2A-Myc-IRESblast* vector (44). These bicistronic or tricistronic vectors express two or three distinct proteins from a single mRNA transcript, driven by the synthetic CAG promoter. The vectors, along with descriptions of cloning strategies, are shown in Table S1.

### mESC culture and generation of stable clones

*Uhrf1*^*−/−*^ mESCs (43) (free from *mycoplasma* contamination) were maintained in gelatin-coated petri dishes without feeder cells in Dulbecco’s Modified Eagle’s Medium with high glucose (Sigma-Aldrich, D5796), supplemented with 15% fetal bovine serum (Sigma-Aldrich, F0926), 0.1 mM nonessential amino acids (Gibco, 11140050), 0.1 mM 2-mercaptoethanol (Sigma-Aldrich, M6250), 50 U/ml penicillin/50 μg/ml streptomycin (Gibco, 15140122), and 1,000 U/ml leukemia inhibitory factor (Millipore, LIF1050). Generation of stable clones by random integration in mESCs was described previously (18, 50). Briefly, *Uhrf1*^*−/−*^ mESCs were transfected with plasmid vectors using Lipofectamine 2000 (Invitrogen, 11668027), then seeded at low density in petri dishes coated with feeder cells, selected with 6 μg/ml of Blasticidin S HCl (Gibco, A1113903) for 7 to 10 days, and individual clones were picked and expanded. Protein expression was analyzed by Western blotting using the following antibodies: UHRF1 (Cell Signaling Technology, #12387, 1:3000), Myc tag (Sigma-Aldrich, M4439, 1:5000), Flag tag (Sigma-Aldrich, F3165, 1:5000), GFP (Santa Cruz Biotechnology, sc-9996, 1:3000), and β-actin (Sigma-Aldrich, A5441, 1:15000). The UHRF1 antibody was validated using *Uhrf1*^*−/−*^ mESCs, the Myc, Flag, and GFP antibodies were validated using tagged proteins expressed in mammalian cell lines, and the β-actin antibody was validated by the vendor.

### DNA methylation assays

DNA methylation at the major and minor satellite repeats in mESCs was analyzed by Southern blotting after digestion of genomic DNA with methylation-sensitive restriction enzymes (*Mae*II for major satellite, *Hpa*II for minor satellite), as described previously (44, 51, 52). DNA methylation levels in the long terminal repeat of IAP retrotransposons in mESCs were measured by bisulfite PCR amplification followed by sequencing, as described previously (52). The biotinylated DNA probes used for Southern blotting and PCR primers used for bisulfite sequencing are shown in Table S2.

DNA methylation analysis of selected CpG sites in *CDX1*, *FOXP3*, *NKX2-6*, and *RASSF1A* promoters and *LINE1* and *ERVK* regions in HCT116 cell lines was performed with bisulfite PCR amplification followed by pyrosequencing methylation analyses (53). Each reaction included controls for high methylation (M.SssI-treated DNA), low methylation *via* whole genome amplification (WGA)-amplified DNA, partial methylation (SW, mixture of M.SssI and WGA), and no-DNA template. DNA methylation values of technical replicates were averaged to report DNA methylation levels per sample, per assay. All steps were performed by the Epigenomics Profiling Core (EpiCore) at The University of Texas MD Anderson Cancer Center (MDACC). The PCR primers for and raw data of pyrosequencing analyses are shown in Table S3.

### RT-qPCR

Total RNA was extracted from human cancer cell lines using TRIzol (Thermo Fisher Scientific, 15596018). Reverse transcription (RT) was performed using iScript Reverse Transcription Supermix (Bio-Rad, 1708841) to generate cDNA. qPCR was performed using iTaq Universal SYBR Green Supermix (Bio-Rad, 1725121) on the CFX Opus 96 Real-Time PCR system (Bio-Rad) using primers specific for *hUHRF1* variants or common for all variants (Table S2), with 18S rRNA serving as internal control. Each sample was analyzed in triplicate, and the average cycle threshold (Ct) values were used to determine the relative levels of *hUHRF1* variants and total *hUHRF1* with the standard 2^−ΔΔC^_T_ method (54).

### CRISPR/Cas9 gene editing

hUHRF1 isoform 2 KO HCT116 cells were generated by CRISPR/Cas9 gene editing as described previously with slight modifications (36). Briefly, a synthetic DNA fragment (C-2970, Table S2) containing the sequence for U6 promoter-driven single-guide RNA (sgRNA) was cotransfected with the *pCAG-Cas9-IRES-GFP* vector into HCT116 cells, and GFP-positive cells were sorted 24 h post-transfection and seeded at low density to derive individual clones. Mutant clones were identified by DNA sequencing.

## Data availability

All data are contained within the manuscript.

## Supporting information

This article contains supporting information.(44), Figures S1 and S2, and Tables S1–S3. One reference was cited in Table S1

## Conflict of interest

The authors declare that they have no conflicts of interest with the contents of this article.
