# Supplementary material for: Functional characterization of UHRF1 variants in facilitating DNA methylation
Source: J Biol Chem. 2026 May 14;302(7):113149. doi: 10.1016/j.jbc.2026.113149 (PMC13264223; doi:10.1016/j.jbc.2026.113149)
Supplement: Supplementary Material [file mmc1.pdf]

Supporting information

Functional characterization of UHRF1 variants in facilitating DNA methylation

Bigang Liu[1], Kaila Nayvelt[1], Swanand Hardikar[1], Kimie Kondo[1], Marcos R. Estecio[1], Xiaodong Cheng[1,2], Taiping Chen[1,2,*]

[1]Department of Epigenetics and Molecular Carcinogenesis, The University of Texas MD Anderson Cancer Center, Houston, Texas 77030, USA

[2]Program in Genetics and Epigenetics, The University of Texas MD Anderson Cancer Center UTHealth Graduate School of Biomedical Sciences, Houston, Texas 77030, USA

*Correspondence: tchen2@mdanderson.org

This file contains:

Figure S1. UHRF1 in different species

Figure S2. Sequence alignment of the linker region between TTD and PHD in human and mouse UHRF1

Table S1. Plasmid vectors for protein expression

Table S2. Synthesized DNA (primers, oligos, probes, and fragments)

Excel document not included in this file:

Table S3. Pyrosequencing

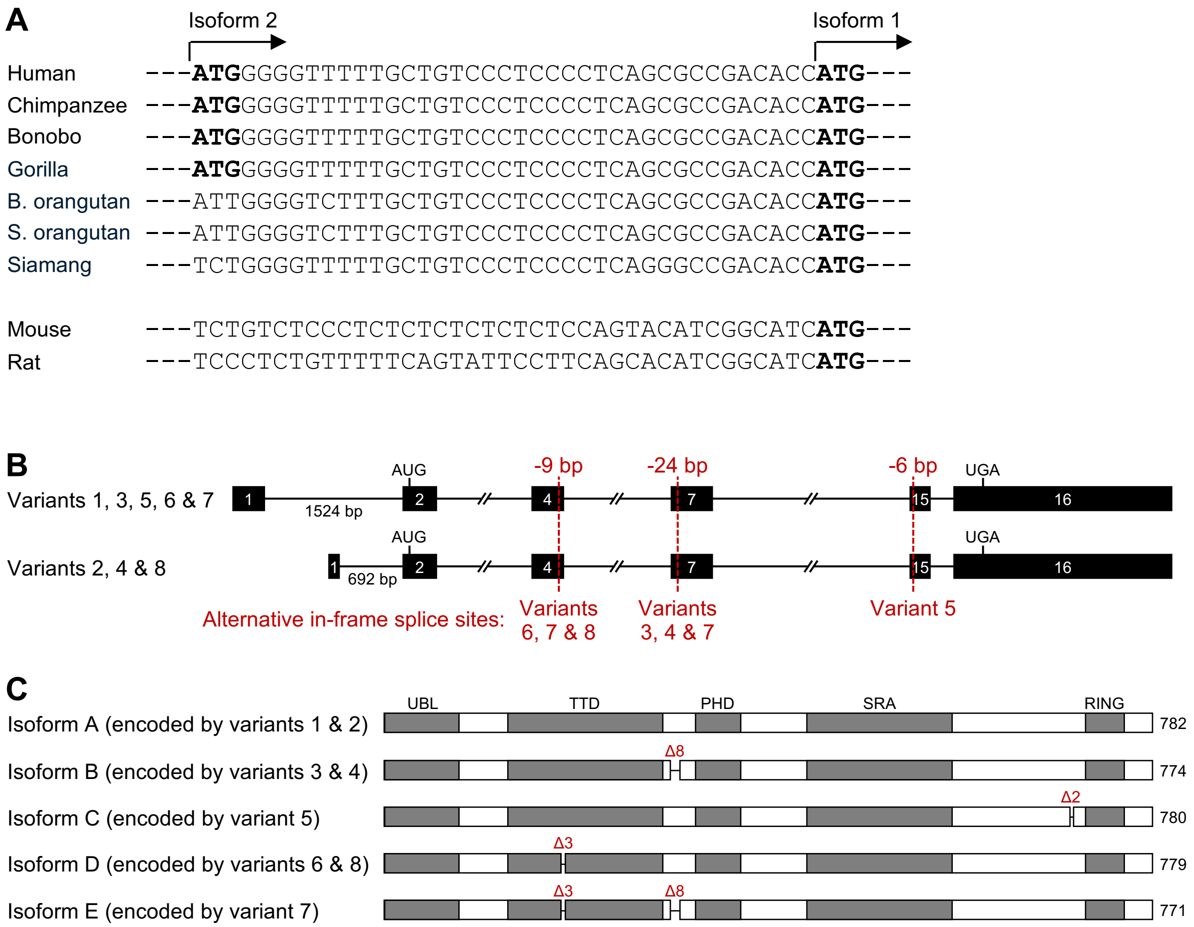

Figure S1. UHRF1 in different species. (**A**) Genomic sequences between the start codons of the two UHRF1 isoforms in several primates and rodents. (**B**) Schematic representation of how the eight *mUhrf1* transcript variants are generated [GenBank accession numbers: NM_010931.4 (variant 1), NM_001111078.2 (variant 2), NM_001111079.2 (variant 3), NM_001111080.2 (variant 4), NM_001413357.1 (variant 5), NM_001413358.1 (variant 6), NM_001413359.1 (variant 7), and NM_001413360.1 (variant 8)]. They are produced by transcription initiated at two different sites and alternative in-frame splicing at the end of exon 4 and/or at the beginning of exon 7 or 15 (splice sites indicated by dashed red lines). The exon/intron organization of *mUhrf1* and *hUHRF1* is highly conserved except that the relatively short intron between exons 12 and 13 in *hUHRF1* is retained in *mUhrf1.* Thus, *mUhrf1* variants comprise 16 exons, rather than 17 that are present in most *hUHRF1* variants. (**C**) mUHRF1 protein products. Isoform A (full-length mUHRF1, consisting of 782 residues) is encoded by variants 1 and 2, and the other isoforms (isoforms B-E) contain 2- to 11-residue in-frame deletions due to the alternative splicing events.

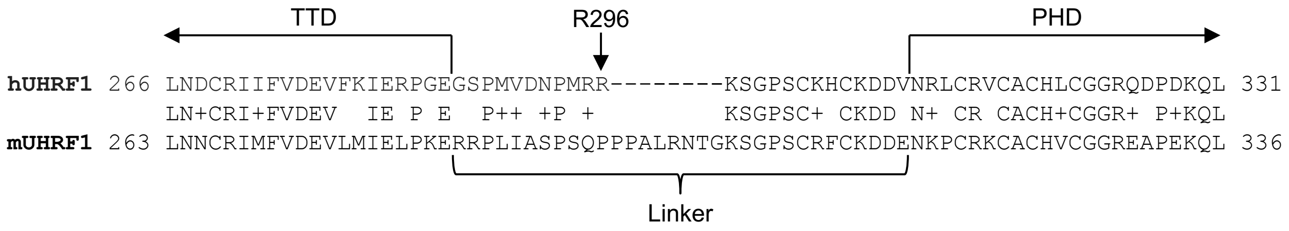

Figure S2. Sequence alignment of the linker region between TTD and PHD in human and mouse UHRF1. Note that R296 in hUHRF1 is not conserved in mUHRF1.

Table S1. Plasmid vectors for protein expression

Vector	Cloning strategy
hUHRF1 (untagged)	• Amplify *hUHRF1* cDNA with primers C-2844 (F) and C-2845 (R).<br>• Clone PCR fragment into *Spe*I-*Eco*RI sites of *pCAG-3xFlag-IRESblast* vector (1). Note: *Spe*I-*Eco*RI digestion removes 3xFlag tag in vector.
hUHRF1:R296W	• Introduce R296W mutation using primers C-2891 (R) and C-2892 (F) in combination with C-2844 (F) and C-2845 (R).<br>• Clone PCR fragment into *Spe*I-*Eco*RI sites of *pCAG-3xFlag-IRESblast* vector.
hUHRF1:R618X<br>(i.e., 1-617 aa)	• Amplify *hUHRF1* fragment with primers C-2844 (F) and C-2893 (R) to delete sequence for C-terminal 176 residues.<br>• Clone PCR fragment into *Spe*I-*Eco*RI sites of *pCAG-3xFlag-IRESblast* vector.
Myc-hUHRF1	• Digest synthesized DNA fragment C-2925 with *Spe*I-*Not*I.<br>• Replace *Spe*I-*Not*I fragment in hUHRF1 vector. Note: *hUHRF1* coding region contains a *Not*I site.
hUHRF1 isoform 2	• Digest synthesized DNA fragment C-2924 with *Spe*I-*Not*I.<br>• Replace *Spe*I-*Not*I fragment in hUHRF1 vector.
mUHRF1 (untagged)	• Amplify *mUhrf1* cDNA with primers C-1426 (F) and C-568 (R).<br>• Clone PCR fragment into *Spe*I-*Eco*RI sites of *pCAG-3xFlag-IRESblast* vector.
Flag-mUHRF1	• Amplify *mUhrf1* cDNA with primers C-567 (F) and C-568 (R).<br>• Clone PCR fragment into *Not*I-*Eco*RI sites of *pCAG-3xFlag-IRESblast* vector.
Myc-mUHRF1(+M)	• Anneal oligos C-2821 (F) and C-2822 (R), creating *Spe*I and *Bam*HI overhangs.<br>• Replace *Spe*I-*Bam*HI fragment in mUHRF1 vector. Note: *mUhrf1* cDNA contains a *Bam*HI site at beginning of coding region.
Myc-mUHRF1(-M)	• Anneal oligos C-2823 (F) and C-2824 (R), creating *Spe*I and *Bam*HI overhangs.<br>• Replace *Spe*I-*Bam*HI fragment in mUHRF1 vector.
mUHRF1-Myc	• Two rounds of PCR to add sequence for Myc tag to mUHRF1 C terminus: 1st round with primers C-1426 (F) and C-2829 (R), 2nd round with primers C-1426 (F) and C-2830 (R).<br>• Clone 2nd round PCR fragment into *Spe*I-*Eco*RI sites of *pCAG-3xFlag-IRESblast* vector.
1A-mUHRF1	• Anneal oligos C-2926 (F) and C-2927 (R), creating *Spe*I and *Bam*HI overhangs.<br>• Replace *Spe*I-*Bam*HI fragment in mUHRF1 vector.
3A-mUHRF1	• Anneal oligos C-2827 (F) and C-2828 (R), creating internal *Not*I site and *Spe*I and *Bam*HI overhangs.<br>• Replace *Spe*I-*Bam*HI fragment in mUHRF1 vector. Note: *Not*I site was subsequently used to generate 7A- and 10A-mUHRF1 vectors.
7A-mUHRF1	• Anneal oligos C-2928 (F) and C-2929 (R), creating *Spe*I and *NotI* overhangs.<br>• Clone into *Spe*I-*Not*I sites of 3A-mUHRF1 vector.
10A-mUHRF1	• Anneal oligos C-2832 (F) and C-2833 (R), creating *Spe*I and *NotI* overhangs.<br>• Clone into *Spe*I-*Not*I sites of 3A-mUHRF1 vector.
hUHRF1-P2A-GFP<br>&<br>hUHRF1:R296W-P2A-GFP	• Amplify cDNA for EGFP with primers C-2910 (F) and C-2911 (R).<br>• Clone PCR fragment into *Bam*HI-*Eco*RI sites of *pCAG-3xFlag-P2A-Myc-IRESblast* vector (1). Note: *Bam*HI-*Eco*RI digestion removes Myc tag in vector.<br>• Amplify cDNA for hUHRF1 or hUHRF1:R296W with primers C-2844 (F) and C-2908 (R).<br>• Clone PCR fragments into *Spe*I-*Not*I sites of vector created in previous step. Notes: a) *Spe*I-*Not*I digestion removes 3xFlag tag in vector; b) *Not*I digestion of PCR products was partial (*hUHRF1* cDNA contains a *Not*I site), and full-length fragments were cloned.
hUHRF1-P2A-hUHRF1:R618X<br>&<br>hUHRF1:R296W-P2A-hUHRF1:R618X	• Amplify cDNA for R618X (i.e., 1-617 aa) with primers C-2909 (F) and C-2983 (R).<br>• Clone fragment into *Bam*HI-*Eco*RI sites of *pCAG-3xFlag-P2A-Myc-IRESblast* vector (1). Note: *Bam*HI digestion of PCR product was partial (*hUHRF1* cDNA contains a *Bam*HI site), and full-length fragment was cloned.<br>• Digest hUHRF1-P2A-GFP and hUHRF1:R296W-P2A-GFP vectors with *Spe*I-*Not*I (partial digestion with *Not*I).<br>• Clone full-length fragments for hUHRF1 or hUHRF1:R296W into *Spe*I-*Not*I sites of vector created in previous step.

Reference:

1. Kim, S. J., Zhao, H., Hardikar, S., Singh, A. K., Goodell, M. A., and Chen, T. (2013) A DNMT3A mutation common in AML exhibits dominant-negative effects in murine ES cells. Blood **122**, 4086-4089.

Table S2. Synthesized DNA (primers, oligos, probes, and fragments)

Name	Sequence (5'-3')	Application
C-2844 (F)	CTAACTAGTCCACCATGTGGATCCAGGTTC	Amplify *hUHRF1* cDNA for hUHRF1 (untagged) vector
C-2845 (R)	TTGGAATTCACCGGCCATTGCCGTAGCCG	
C-2891 (R)	CCGCTCTTCCaTCTCATGGGGTTGTCAAC	Introduce hUHRF1:R296W mutation
C-2892 (F)	ACCCCATGAGAtGGAAGAGCGGGCCGTCCT	
C-2893 (R)	TCGAATTCaGTTGGCCAGGGCTTCCAGGTAG (used with C-2844)	For hUHRF1:R618X vector
C-2924	CTAGAACTAGTCCACC**ATGGGCGTGTTCGCTGTGCCTCCACTCAGCGCCGATACT**ATGTGGATTCAGG<br>TTCGGACCATGGACGGGAGGCAGACCCACACGGTGGACTCGCTGTCCAGGCTGACCAAGGTGGAGGAG<br>CTGAGGCGGAAGATCCAGGAGCTGTTCCACGTGGAGCCAGGCCTGCAGAGGCTGTTCTACAGGGGCAA<br>ACAGATGGAGGACGGCCATACCCTCTTCGACTACGAGGTCCGCCTGAATGACACCATCCAGCTCCTGG<br>TCCGCCAGAGCCTCGTGCTCCCACACAGCACCAAGGAGCGGGACTCCGAGCTCTCCGACACCGACTCC<br>GGCTGCTGCCTGGGCCAGAGTGAGTCAGACAAGTCCTCCACCCACGGTGAGGCGGCCGCCGAGA	Synthesized DNA fragment for hUHRF1 isoform 2 vector (sequence coding 13-residue N-terminal extension in bold)
C-2925	CTAGAACTAGTCCACCATG**GAACAAAAGCTGATTAGCGAAGAGGACCTG**ATGTGGATCCAGGTTCGGA<br>CCATGGACGGGAGGCAGACCCACACGGTGGACTCGCTGTCCAGGCTGACCAAGGTGGAGGAGCTGAGG<br>CGGAAGATCCAGGAGCTGTTCCACGTGGAGCCAGGCCTGCAGAGGCTGTTCTACAGGGGCAAACAGAT<br>GGAGGACGGCCATACCCTCTTCGACTACGAGGTCCGCCTGAATGACACCATCCAGCTCCTGGTCCGCC<br>AGAGCCTCGTGCTCCCCCACAGCACCAAGGAGCGGGACTCCGAGCTCTCCGACACCGACTCCGGCTGC<br>TGCCTGGGCCAGAGTGAGTCAGACAAGTCCTCCACCCACGGTGAGGCGGCCGCCGAGA	Synthesized DNA fragment for Myc-hUHRF1 vector (sequence coding Myc tag in bold)
C-1426 (F)	CTAACTAGTCCACCATGTGGATCCAGGTTCGAACT	Amplify *mUhrf1* cDNA for mUHRF1 (untagged) vector
C-568 (R)	CTCGAATTCACCGGCCGCTGCCATAGCCAG	
C-567 (F)	ATCGCGGCCGCGTGGATCCAGGTTCGAACTATGG (used with C-568)	For Flag-mUHRF1 vector
C-2821 (F)	CTAGTCCACCATGGAACAAAAGCTGATTAGCGAAGAGGACCTGATGTG	Oligos annealed for generating Myc-mUHRF1(+M) vector
C-2822 (R)	GATCCACATCAGGTCCTCTTCGCTAATCAGCTTTTGTTCCATGGTGGA	
C-2823 (F)	CTAGTCCACCATGGAACAAAAGCTGATTAGCGAAGAGGACCTGTG	Oligos annealed for generating Myc-mUHRF1(-M) vector
C-2824 (R)	GATCCACAGGTCCTCTTCGCTAATCAGCTTTTGTTCCATGGTGGA	
C-2829 (R)	TCGCTAATCAGCTTTTGTTCCCGGCCGCTGCCATAGCCAG (used with C-1426)	Reverse primers used for mUHRF1-Myc vector
C-2830 (R)	CTCGAATTCACAGGTCCTCTTCGCTAATCAGCTTTTGTTC (used with C-1426)	
C-2926 (F)	CTAGTCCACCATGGCTTG	Oligos annealed for generating 1A-mUHRF1 vector
C-2927 (R)	GATCCAAGCCATGGTGGA	
C-2827 (F)	CTAGTCCACCATGGCGGCCGCTTG	Oligos annealed for generating 3A-mUHRF1 vector
C-2828 (R)	GATCCAAGCGGCCGCCATGGTGGA	
C-2928 (F)	CTAGTCCACCATGGCTGCAGCTGCTGC	Oligos annealed for generating 7A-mUHRF1 vector
C-2929 (R)	GGCCGCAGCAGCTGCAGCCATGGTGGA	
C-2832 (F)	CTAGTCCACCATGGCTGCAGCTGCTGCAGCAGCTGC	Oligos annealed for generating 10A-mUHRF1 vector
C-2833 (R)	GGCCGCAGCTGCTGCAGCAGCTGCAGCCATGGTGGA	
C-2910 (F)	TATGGATCCATGGTGAGCAAGGGCGAG	Amplify *EGFP* cDNA for hUHRF1-P2A-GFP vectors
C-2911 (R)	CGCGAATTCTACTTGTACAGCTCGTCC	
C-2909 (F)	TAATAGGATCCATCCAGGTTCGGACCATG (used with C-2983)	Amplify hUHRF1:R618X for hUHRF1-P2A-R618X vectors
C-2932 (F)	CACCATGTGGATCCAGGTTCG	*hUHRF1* RT-qPCR (common for all variants)
C-2933 (R)	TCATTCAGGCGGACCTCGTAG	
C-3000 (F)	AGACAAGCTGTTCGCGGCGAC (used with C-2933)	RT-qPCR (variant 1 specific)
C-2931 (F)	CTCAGAGGTGCTGGTAAAACTG (used with C-2933)	RT-qPCR (variant 2 specific)
C-3001 (F)	TTCCAGCGATTCTCCCACCTC (used with C-2933)	RT-qPCR (variant 3 specific)
C-3002 (F)	ACGCCGAGGGTCCAGGGTTTG (used with C-2933)	RT-qPCR (variant 4 specific)
C-3003 (F)	TTCTGGGATTCCCTCTTCCCG (used with C-2933)	RT-qPCR (variant 5 specific)
C-1564	TTAGAAATGTCCACTGTAGGACGTGGAATATGGCAAG	Biotinylated major satellite probe
C-1565	TGGAAACGGGATTTGTAGAACAGTGTATATCAATGAG	Biotinylated minor satellite probe
C-462 (F)	CACGGATCCTTGTGTTTTAAGTGGTAAATAAAT	Primers for bisulfite sequencing analysis of IAP LTR
C-464 (R)	GTCGAATTCAAAAAAAACACACAAACCAAAAT	
C-2970	GAATTAACCCTCACTAAAGGGACAATTCAGGTACCAAGGTCGGGCAGGAAGAGGGCCTATTTCCCATG<br>ATTCCTTCATATTTGCATATACGATACAAGGCTGTTAGAGAGATAATTAGAATTAATTTGACTGTAAA<br>CACAAAGATATTAGTACAAAATACGTGACGTAGAAAGTAATAATTTCTTGGGTAGTTTGCAGTTTTAA<br>AATTATGTTTTAAAATGGACTATCATATGCTTACCGTAACTTGAAAGTATTTCGATTTCTTGGCTTTA<br>TATATCTTGTGGAAAGGACGAAACACCG**GGTGCTGGTAAAACTGATGG**GTTTTAGAGCTAGAAATAGC<br>AAGTTAAAATAAGGCTAGTCCGTTATCAACTTGAAAAAGTGGCACCGAGTCGGTGCTTTTTTTCTAGA<br>CCCAGCTGCCCTATAGTGAGTCGTATTACG	Synthesized DNA for CRISPR/Cas9 gene editing to generate hUHRF1 isoform 2 KO HCT116 cells (targeting sequence in bold)
C-2937 (F)	AGGGTCTGGCTGTACAGGAG	Primers for screening hUHRF1 isoform 2 KO clones
C-2938 (R)	GTAGAACAGCCTCTGCAGGC	

F, forward; R, reverse. Restriction sites used for cloning are underlined. Mutations in primers are shown in lower case.
